# The Role of the Substantia Nigra Pars Compacta in Regulating Sleep Patterns in Rats

**DOI:** 10.1371/journal.pone.0000513

**Published:** 2007-06-06

**Authors:** Marcelo M.S. Lima, Monica L. Andersen, Angela B. Reksidler, Maria A.B.F. Vital, Sergio Tufik

**Affiliations:** 1 Departamento de Psicobiologia, Universidade Federal de São Paulo, São Paulo, Brazil; 2 Departamento de Farmacologia, Universidade Federal do Paraná, Curitiba, Brazil; Emory University, United States of America

## Abstract

**Background:**

As of late, dopaminergic neurotransmission has been recognized to be involved in the generation of sleep disturbances. Increasing evidence shows that sleep disturbances in Parkinson's disease (PD) patients are mostly related to the disease itself, rather than being a secondary phenomenon. Evidence contained in the literature lends support to the hypothesis that the dopaminergic nigrostriatal pathway is closely involved in the regulation of sleep patterns.

**Methodology/Principal Findings:**

To test this hypothesis we examined the electrophysiological activity along the sleep-wake cycle of rats submitted to a surgically induced lesion of the SNpc by 1-methyl-4-phenyl-1,2,3,6-tetrahydropyridine (MPTP). We demonstrated that a 50% lesion of the substantia nigra pars compacta (SNpc) suffices to produce disruptions of several parameters in the sleep-wake pattern of rats. A robust and constant decrease in the latency to the onset of slow wave sleep (SWS) was detected throughout the five days of recording in both light [F_(22.16)_ = 72.46, p<0.0001] and dark [F_(22.16)_ = 75.0, p<0.0001] periods. Also found was a pronounced increase in the percentage of sleep efficiency during the first four days of recording [F_(21.15)_ = 21.48, p<0.0001], in comparison to the sham group. Additionally, the reduction in the SNpc dopaminergic neurons provoked an ablation in the percentage of rapid eye movement sleep (REM) during three days of the sleep-wake recording period with a strong correlation (r = 0.91; p<0.0001) between the number of dopaminergic neurons lost and the percentage decrease of REM sleep on the first day of recording. On day 4, the percentage of REM sleep during the light and dark periods was increased, [F_(22.16)_ = 2.46, p<0.0007], a phenomenon consistent with REM rebound.

**Conclusions/Significance:**

We propose that dopaminergic neurons present in the SNpc possess a fundamental function in the regulation of sleep processes, particularly in promoting REM sleep.

## Introduction

Parkinson's disease (PD) is a neurodegenerative disorder mainly characterized by motor deficits generated by the decrease of dopaminergic neurons within the substantia nigra pars compacta (SNpc). PD patients endure severe sleep disturbances, such as excessive daytime sleepiness [1], rapid eye movement sleep (REM) behavior disorders [2], “sleep attacks” [3] and cessation of dream recall [4] while L-DOPA therapy has been found to enhance vivid dream recall [5]. Recent studies show increasing evidence that the disturbance of sleep and wakefulness in PD is most intimately related to the disease itself and does not exclusively represent a secondary phenomenon [6], [7]. This primary effect of PD on sleep may be linked to the previously recognized [8], although only recently fully appreciated, role of dopamine (DA) in modulating sleep-wake states [7].

PD is a disease characterized by impairment of the nigrostriatal pathway. This pathway is known to control and initiate motor plans, from the dorsal striatum to the motor cortex and back to the cortex via the thalamus [9]. Patients with PD who have extensive loss of dopaminergic cells within the SNpc, and less so within the ventral tegmental area, often have increased sleepiness, which is made worse by the presence of dopaminergic D_2_ receptor agonists [10], [11]. Such involvement of DA has been described subsequent to sleep deprivation as being directly involved in the generation of dopaminergic D_2_ receptor supersensitivity [12], [13], [14]. Moreover, recent findings demonstrate that partial DA depletion causes disturbances of REM sleep without affecting motor functions [15]. Additionally, a robust increase in the electrophysiological activity of dopaminergic neurons of the ventral tegmental area has been identified during REM sleep [16]. Along with the influence of dopaminergic neurotransmission on generating sleep disturbances, the evidence further supports the hypothesis that the dopaminergic nigrostriatal pathway, specifically, is fundamental in the regulation of sleep patterns. To test this hypothesis we examined the electrophysiological activity along the sleep-wake cycle of rats submitted to a surgically induced lesion of the SNpc induced by 1-methyl-4-phenyl-1,2,3,6-tetrahydropyridine (MPTP). Bilateral guide canullas were implanted in the rats, allowing for the microinjection of the neurotoxin directly into the SNpc. Once microinjected, MPTP is converted, by monoamine oxidase-B in glial cells, to 1-methyl-4-phenylpyridinium (MPP^+^) [17], which is taken up via the neuronal dopamine (DA) transporter and accumulates in dopaminergic neurons. MPP^+^ then becomes concentrated in mitochondria where it inhibits complex Ι reducing ATP generation and causing increased free-radical production and subsequent neuronal death [18]. Intranigral MPTP administration produces a burst of dopaminergic neuronal death, and also a specific modulation in the tyrosine hydroxylase (TH) protein expression within the substantia nigra (SN) [19], [20]. In view of these considerations we sought to determine the fluctuations of TH protein expression within the SN, concomitantly to the sleep-wake pattern, in order to examine the participation of this protein in mediating events that regulate the sleep-wake cycle.

## Materials and Methods

### Subjects

All experiments were conducted in accordance with National Institutes of Health (USA) guidelines for the care and use of animals and abided by an approved animal protocol from our university's ethical committee for animal experimentation (#0737/06). Male wistar rats weighing 280–320 g at the beginning of the experiments were used. Rats were housed individually in transparent acrylic cages and maintained in standard laboratory conditions (22±2°C, 12 h light/dark cycle, lights on 7:00 A.M.) with food and water provided *ad libitum*. Maximal efforts were employed to reduce the number of animals used in the experiments yet enough to ensure unambiguous and reliable statistical analysis and data interpretation.

### Stereotaxic surgery

Rats were distributed at random into the two groups named sham and MPTP. Animals were anesthetized with diazepam (10 mg/kg i.p.) and ketamine (90 mg/kg i.p.) and they were mounted in a classical stereotaxic frame (Insight Instruments). Body temperature was maintained at 37°C with a regulated electric heating pad (Harvard Apparatus). Two bipolar electrodes with four stainless-steel screws (Ø 0.9 mm) were placed into the skull through small holes bored into the right lateral fronto-parietal (one pair) and in the left medial fronto-parietal (another pair) in order to monitor bipolar electroencephalogram (EEG). The free ends of the electrodes were soldered to a socket that was attached to the skull with acrylic dental cement. Two nickel-chromium flexible wires were inserted into the neck muscles in order to record the electromyogram (EMG). Additionally, the animals were implanted guide cannulae bilaterally (20 mm×0.6 mm) 2.0 mm above the SNpc according to the following coordinates: anteroposterior (AP): −5.0 mm from the bregma; mediolateral (ML): ±2.1 mm from the midline; dorsoventral (DV): −7.8 mm from the skull [21]. All the rats received penicillin (20,000 U in 0.1 ml, i.m.) and sodium diclofenac (25 mg/ml, i.p.) after surgery. One week after surgery, the sockets were connected via flexible recording cables and a commutator to a polygraph and computer. After three days with the cables and the cannulae implants, the rats progressively habituated to the apparatus allowing the performance of the 48h basal sleep-wake states recording.

### Intranigral microinjections of MPTP

**Figure 1:**
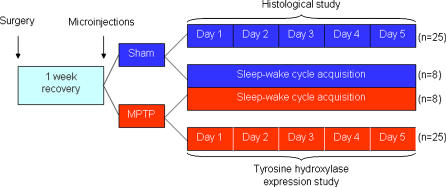
Schematic representation of the experimental design. After the intranigral microinjections of saline or MPTP, the rats were distributed for sleep-wake cycle recording (n = 8/group), midbrain TH immunohistochemical examination (n = 5) and nigral TH protein expression study (n = 5). Histological and western blotting experiments used animals that had their brains collected along the 5 days of recording (n = 5/day/group) at the same hour they were lesioned, according to the time-points schedule.

After the basal sleep-wake recording of states the animals were manipulated and gently immobilized in order to perform the neurotoxin microinjection. Microinjections were performed during the early part of the light period (7:00 A.M. to 9:00 A.M). Each infusion was performed with a 30-gauge stainless injection needle bilaterally introduced in the guide cannulae through which 2 µL of MPTP (100 µg/µL, Sigma, prepared in sterile saline 0.9%,) were administered. The control of the flow of the microinjection was made by using an electronic pump (Insight Instruments) at a rate of 0.40 µL/min for 2.5 min, followed by 2 min with the needle in the injection site to avoid reflux. Sham microinjections followed the same procedure but using 2 µL of sterile saline 0.9%. Immediately after the microinjection procedure, the rats were placed in their home cages and the data acquisition initiated for a period up to 5 days (for details of the experimental protocol see Fig. 1).

### Sleep-wake state identification

Electrophysiological signals were recorded on a digital polygraph (Neurofax QP 223A ©Nihon Kohden) at a sampling rate of 200 Hz. Filter settings of EEG were at 35 Hz and the time constant was 0.1 s. For the EMG the filter setting was at 70 Hz and the time constant was 0.03 s. EEG and EMG signals were calibrated at 50 µV pulses. In homeothermic animals, sleep was divided into two main distinct stages: slow wave sleep (SWS) and REM sleep. The former is characterized by high-voltage slow oscillations in the EEG associated with weak EMG activity. During REM sleep, activity in the forebrain is comparable to waking levels with pronounced and sustained theta rhythm in the hippocampus and a complete loss of muscle tone [22], [23]. Recordings of epochs were displayed at 15 s intervals on a high resolution PC monitor and visually classified as wakefulness (W), SWS and REM sleep.

### TH immunohistochemistry of the SNpc neurons

For the immunohistochemical study of neuronal dopaminergic population within the SNpc, rats were deeply anesthetized with ketamine and intracardially perfused with saline and 4% of the fixative solution of formaldehyde in 0.1 M phosphate buffer (pH 7.4) during the period the sleep wake states were recorded, which amounted to five days. Brains were removed from the skulls and were immersed for 1 week in that fixative solution at 4°C. Subsequently, the brains were placed in 30% sucrose solution for 48h before sectioning. Series of 30 µm thick sections were cut on a cryostat in the frontal plane and collected at −0.48 mm to −0.56 mm from the bregma [21]. Tissue sections were incubated with primary antibody anti-TH, raised in rabbits, diluted in PBS containing 0.3% Triton X-100 (1∶500; cat #AB152 Chemicon) and reacted overnight at 4°C. Biotin conjugated secondary antibody incubation (1∶200 cat #S-1000 Vector Laboratories), was performed for 2 h at room temperature. After several washes in PBS, antibody complex was localized using the ABC system (Vectastain ABC Elite kit cat #PK6101, Vector Laboratories) followed by 3,3′-diaminobenzidine reaction with nickel enhancement. The sections were then mounted onto gelatin-coated slides and coverslipped after dehydration in ascending concentrations of ethanol-xylene solutions.

### Quantification of dopaminergic neurons

Unbiased quantification of TH-labeled neurons from the SNpc relied on the software Freeware NIH Image, 1.63. Counts were done on 8–10 tissue sections (one in four series), and an average count per section was determined for each animal. The selected areas were digitized with a digital camera DP71 (Olympus Optical) using an Olympus microscope BX50.

### Determination of TH protein expression

To determine TH expression a different set of animals underwent the same protocol of stereotaxic surgery, cannulae implantation, and lesioning with MPTP microinjection. To investigate the TH expression fluctuations, generated by MPTP among the sleep-wake cycle, 10 rats (5 of sham group and 5 of MPTP group) were killed daily, by decapitation, at the same time of the MPTP microinjection. After the decapitation the brains were quickly dissected with the isolation of the SN which was immediately frozen in dry ice and stored at −80°C until the lysis procedure. The lysis were performed in 1.5 mL eppendorf tubes by sonication in the presence of an ice-cold buffer containing 50 mM Tris (pH 8.0), 250 mM NaCl, 1% NP-40, 0.1% sodium dodecyl sulfate (SDS), 0.25% sodium deoxycholate, 2 mM EDTA, 1mM dithiothreitol (DTT), 20 µM phenylmethylsulfonyl fluoride (PMSF), and protease inhibitors (Complete tablet; Roche). After incubation on ice for 30 min extracts were centrifuged at 12,000× g for 40 min at 4°C, and the supernatants for protein extracts were collected and stored at −80°C for further western blotting analysis. The aliquot of supernatant was collected for total protein analysis [24]. Samples containing equal amounts of total protein (5 µg per lane) were boiled with SDS sample buffer and electrophresed in 10% SDS-polyacrylamide gels in a Mini Protean II Dual Slab Cell (Bio-Rad). Proteins were electrophoretically transferred to nitrocellulose membranes by means of a Mini transblot electrophoretic transfer cell (Bio-Rad). Each membrane was blocked for 1 h in 10% nonfat dry milk/0.5% Tween-20 in Tris-buffered saline. Subsequently each membrane was probed overnight at 4°C with mouse monoclonal antibodies against TH (1∶5,000; cat #T2928 Sigma) or β-tubulin ΙΙΙ (1∶500; cat #MAB1637 Chemicon) followed by several washes in TBST and incubation with an adequate secondary horseradish peroxidase-conjugated antibody (1∶5,000; cat #30021019 GE) for 60 min, and visualized by chemiluminescence (cat #sc 2048 Santa Cruz Biotechnology). The bands were quantified by using the software ImageJ 1.32j.

### Statistical methods

Differences in number of cell counts and TH protein expression data underwent analysis of variance (ANOVA) followed by the Newman-Keuls test. Sleep parameters were analyzed by ANOVA and the Tukey test was used as *post hoc* when indicated. Pearson correlation coefficients were calculated for comparison of loss of dopaminergic neurons or TH protein expression with alterations in sleep parameters. Differences were considered significant if p<0.05. The values were expressed as mean±S.E.M.

## Results

### Characterization of dopaminergic neuronal loss

**Figure 2:**
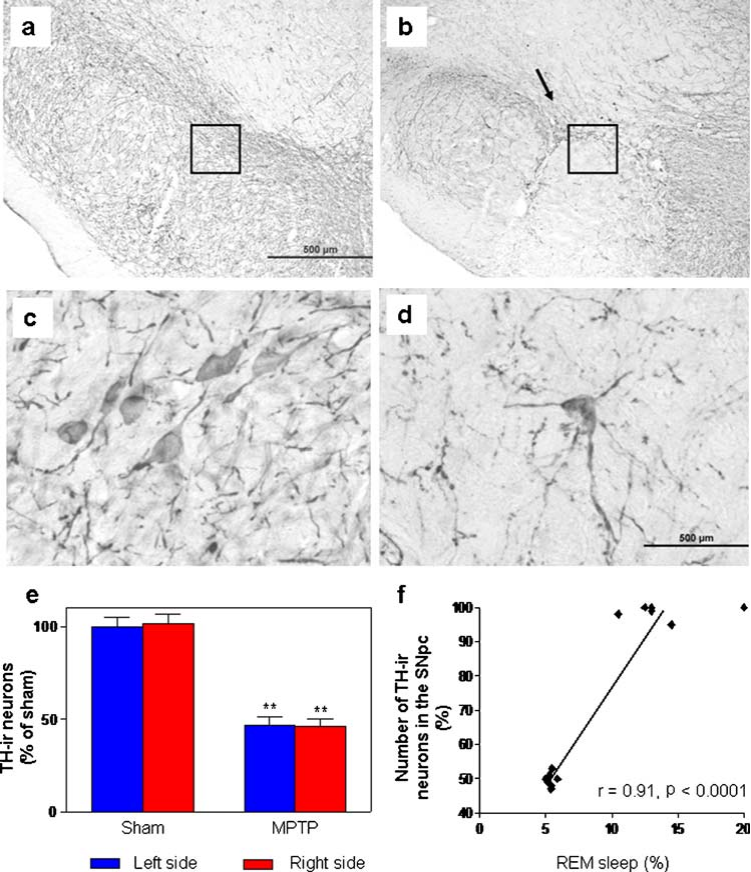
Dopaminergic neurons present in the SNpc are reduced by half after MPTP intranigral microinjection. A representative immunohistochemistry labeling of TH-ir neurons is shown in animals at the end of 5-day sleep-wake cycle recordings; (a) sham group (the inset square shows the specific region depicted in the panel below), (b) MPTP group (the inset square shows the specific region depicted in the panel below), (c) sham group in higher magnification (d) MPTP group in higher magnification, (e) bilateral quantification of the loss of TH-ir neurons in the SNpc, (f) the loss of TH-ir neurons in the SNpc correlated closely with the decrease of REM sleep on the first day of recording, subsequent to MPTP microinjection. The values are expressed as mean±S.E.M. **p<0.01, ANOVA followed by the Newman–Keuls test.

Representative photomicrographs of TH immunohistochemistry in the SNpc are shown for both sham and MPTP groups (Fig. 2). The TH-ir neurons were prominently detectable in the SNpc of sham rats. The bodies and fibers of dopaminergic neurons showed intense staining with evident immunopositive processes in the sham group (Fig. 2a,c). The mean number of TH-ir neurons within the SNpc (sham, 9,750±345 vs MPTP, 4,873±176) indicated that MPTP inflicted an expressive dopaminergic neuronal loss of 50% [F_(10.47)_ = 17.25, p<0.01] restricted to the SNpc (Fig. 2e), with no detectable damages in the ventral tegmental area.

### Effects of dopaminergic cell loss in the SNpc upon sleep-wake cycles

**Figure 3:**
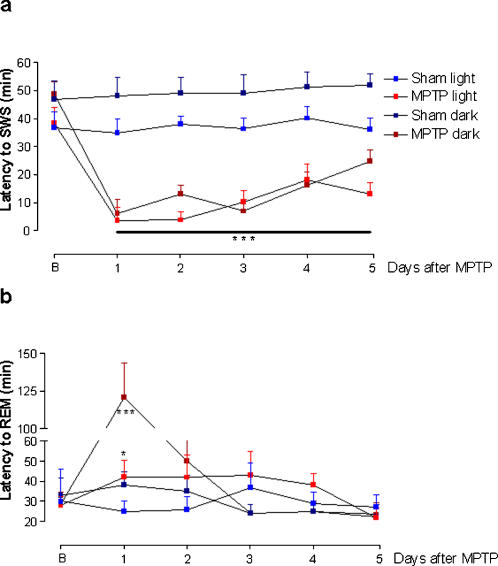
Dual effects upon the latencies to SWS and REM sleep after selective SNpc lesion. (a) Latency to SWS showed to be decreased at all time-points after MPTP exposure, in both light and dark periods. (b) Latency to REM sleep was increased in the light and dark periods after MPTP only on the first day of recording. The values are expressed as mean±S.E.M. *p<0.05, ***p<0.0001 compared to baseline, ANOVA followed by the Tukey test.

**Figure 4:**
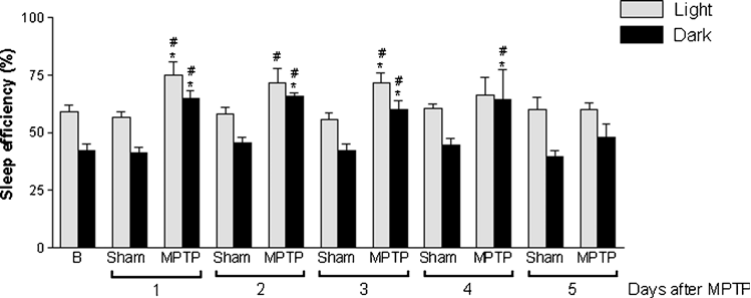
Nigral disruption promoted an increase in the sleep efficiency during the first four days of recording. SNpc dopaminergic neuronal loss promoted a sustained increase in the percentage of sleep efficiency on the first 3 days of the sleep-wake recording, in both light and dark periods. On the fourth day, the increase in this parameter occurred only in the dark period. The values are expressed as mean±S.E.M. **p<0.05 compared to baseline, ^#^p<0.05 compared to the respective sham group, ANOVA followed by the Tukey test.

**Figure 5:**
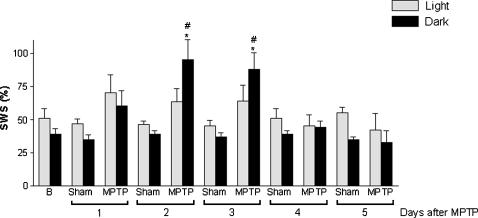
Slight increase in the percentage of SWS after SNpc lesion. The percentage of SWS was increased on the second and fourth days, only in the dark periods, indicating sleepiness effect in the activity period of the rodent. The values are expressed as mean±S.E.M. *p<0.05 compared to those of baseline, ^#^p<0.05 compared to the respective sham group. ANOVA followed by the Tukey test.

**Figure 6:**
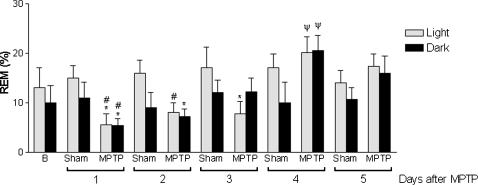
SNpc neurons are fundamental in the generation of REM sleep. The percentage of REM sleep, of the MPTP group, was reduced on the first three days of recording, in both light and dark periods. In contrast, the percentage of REM sleep significantly rose on the fourth day, probably as a compensatory rebound mechanism. The values are expressed as mean±S.E.M. *p<0.05 compared to those of baseline, ^#^p<0.05 compared to the respective sham group, ^ψ^p<0.0007 compared to the MPTP group day 1. ANOVA followed by the Tukey test.

The data indicated that a 50% dopaminergic neuronal loss restricted to the SNpc, inflicted by MPTP, was able to produce a robust and constant decrease in the latency to the onset of SWS during the 5 days of recording in both light [F_(22.16)_ = 72.46, p<0.0001] and dark [F_(22.16)_ = 75.0, p<0.0001] periods (Fig. 3a). In addition, this nigral dopaminergic depletion also increased the latency to REM sleep during the light [F_(22.16)_ = 14.73, p<0.001] and dark [F_(22.16)_ = 14.70, p<0.0001] periods 1 day after neurotoxin microinjection (Fig. 3b). MPTP-induced dopaminergic neuronal loss also generated pronounced increase in the percentage of sleep efficiency during the first 4 of the 5 days of recording [F_(21.15)_ = 21.48, p<0.0001] in comparison to the sham group (Fig. 4). The percentage of SWS was increased in the MPTP group on days 2 and 3 only in the dark period [F_(21.15)_ = 4.54, p<0.0001] compared to the sham group (Fig. 5). In contrast, the reduction in the dopaminergic neuronal population, resident in the SNpc provoked an ablation in the percentage of REM sleep during days 1 (for light and dark periods), 2 and 3 (for the light period only) (Fig. 6). Moreover, there was a strong correlation between the number of TH-ir neurons lost and the percentage decrease of REM sleep on the first day of recording after MPTP lesion (r = 0.91; p<0.0001, Fig. 2f). On day 4, REM sleep presented an increase in both periods [F_(22.16)_ = 2.46, p<0.0007] (Fig. 6). All the sleep parameters return to basal values at day 5 after the MPTP exposure, with the exception of latency to SWS. This observation indicated the end point of the examination paradigm.

### Fluctuations in the nigral TH protein expression after MPTP microinjection

**Figure 7:**
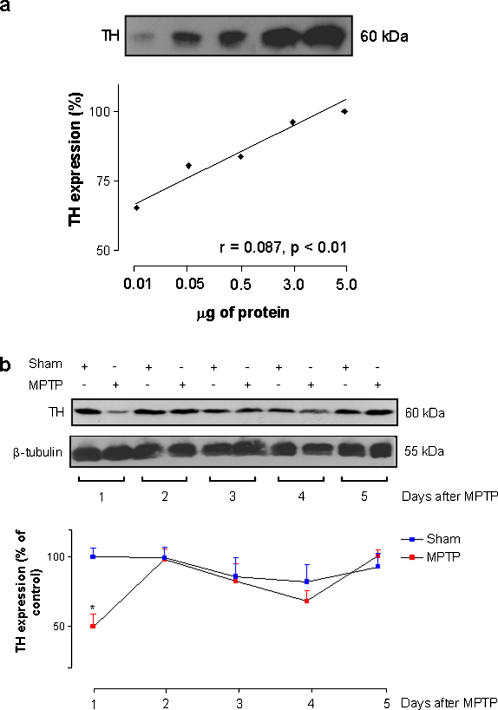
Western blotting analysis of the Nigral TH protein expression along the sleep-wake cycle recording. (a) Serial dilution of nigral TH protein was loaded across the lanes and visualized with enhanced chemiluminescence. A standard curve was generated from densitometric values obtained from computer analysis of digitized film from immunoblot. A linear relationship between optical density and the amount of protein was observed with a strong correlation. (b) TH protein expression was significantly decreased in the MPTP group in comparison to the sham on the first day after the microinjection. Two days after MPTP, TH presented an expression of 98% in comparison to the sham group, on the respective day. A similar situation was observed for the subsequent time-points analyzed. The values are expressed as mean±S.E.M. *p<0.05 compared to those of baseline. ANOVA followed by the Newman-Keuls test.

A single band was observed at the expected molecular mass of 60 kDa for the groups tested at the distinct time-points examined. Denser TH-positive bands were consistently observed in the sham group (Fig. 7a,b). There was a significant decrease of 50.1% of TH expression in the SN of the MPTP group in comparison to the sham group [F_(9.99)_ = 2.95, p<0.004] on the first day after the neurotoxin microinjection (Fig. 7b). This dramatic decrease in TH expression at day 1 was not seen in later time-points. Two days after the microinjection TH expression in the MPTP group was 98% in comparison to the sham group of the respective day. A similar situation was observed for the following time-points: 3 (p = 0.07, 86%), 4 (p = 0.06, 82%) and 5 (p = 0.11, 100%) days after the microinjections. Weak correlations were observed between TH protein expression and sleep parameters (sleep efficiency r = −0.64, p = 0.042; SWS r = −0.30, p = 0.39; REM r = 0.20, p = 0.57) along the time-points.

## Discussion

The main findings herein are that the dopaminergic neurons located within the SNpc played an important role in regulating sleep patterns in rats, and that disturbances in this particular neuronal population produced severe complications in all the sleep parameters examined, especially in REM sleep. To the extent of our knowledge this is the first paper that provides direct evidence of SNpc function in sleep. The impairment in REM sleep presented a transient characteristic which culminated a few days after the nigral disruption with a strong increase in the percentage of REM sleep, during the light and dark periods. The present findings dovetail with growing evidence showing the influence of DA in the regulation of sleep-wake pattern and the significant repercussions that eventual reductions of this neurotransmitter may generate, particularly in PD patients.

A recent experiment has shown a coordinated bursting mode of activity of dopaminergic neurons, located in the ventral tegmental area, initiated 10–20 s before the onset of REM sleep and lasting until the end of this sleep stage [16]. It has been proposed that during the sleep-wake cycle the temporal pattern of firing rates alters in midbrain dopaminergic cells. The available evidence tends to indicate that during wakefulness there occurs an increase of burst firing activity of dopaminergic neurons, and enhanced release of DA in the nucleus accumbens, and a number of forebrain structures [25]. Hence on the basis of our findings, different features of the dopaminergic neurons are revealed, considering that they are not exclusively related to wakefulness as initially postulated, according to previous lesion studies [26], [27]. Nevertheless, a large range of different cell populations are damaged with the systemic administration of a neurotoxin or even by electrolytic protocols. In this sense, the present study promoted a dopaminergic lesion compatible with the extension of the area that was examined, i.e. the SNpc. We adopted the intranigral microinjection of MPTP as a more reliable and accurate choice to produce only specific dopaminergic neuronal death. This protocol resorts to precise delivery of MPTP to the SNpc, relying on its specificity to the dopaminergic transporter. The TH immunohistochemistry examination revealed that MPTP produced a reduction of 50% in the dopaminergic neurons in the SNpc. In addition, neuronal morphology was apparently preserved in the remaining neurons of the MPTP group, despite the remarkable apoptosis inflicted by MPTP [28], [29]. At this point, a note of caution should be added: MPTP microinjection, in our experience, can achieve a plateau of dopaminergic neuronal loss of around 50%, without affecting adjacent areas. In this sense, our hypothesis is limited exclusively to the influence of SNpc neurons in the patterns of the sleep-wake cycle. To ensure an unbiased lesion protocol, we preferred not to produce a more extensive lesion, guaranteeing that the physiological effects occurred only as consequence of SNpc lesion.

The electrophysiological data indicated that the absence of half of the dopaminergic neurons within the SNpc provoked a major impairment in the sleep-wake parameters. Some manifestations, as indicated by the decrease in the latency to SWS, initiated almost immediately after the neurotoxin microinjection, corroborating a previous study with systemic administration of MPTP in cats [30], and those alterations were continuously identified until the end of the experimentation. In relation to these findings, it is noteworthy to consider that the impairments of these parameters could be characterized as an overwhelming episode of sleep, similar to “sleep attacks” which is a manifestation experienced by 1–21% of PD patients [3], [31]. We also demonstrated that latency to REM sleep presented a punctual augment on the very first day after the nigral lesion, with a return to baseline values on the subsequent days of recording. This finding is strengthened by the dramatic reduction in the percentage of REM sleep observed in the first day after MPTP, which is highly correlated to the number of dopaminergic TH-ir neurons lost within the SNpc. Otherwise, TH protein expression in the SN did not present significant correlation to any recorded sleep parameter. Such evidence indicates that sleep-wake patterns are more closely related to function of nigral dopaminergic neurons than exclusively to variations of TH expression.

An analogous pattern of deficit in REM sleep was observed the following days, with some level of fluctuation in the percentage of REM sleep although on the 4^th^ day of recording, REM sleep robustly increased in both light and dark periods. Such unexpected result suggests the activation of a compensatory mechanism which led to an irrefutable augmentation in the percentage of REM sleep. It is not unreasonable to suggest that this phenomenon can be considering a rebound of REM sleep.

The dopaminergic nigrostriatal lesion promoted a significant reduction in the TH protein expression, and suggestively in DA biosynthesis. Nevertheless, 50% of the dopaminergic nigral neurons survived generating a potential plastic response to this neurotoxic assault. This response is possibly mediated by the TH protein up-regulation and also by an important post-synaptic event defined as D_2_ dopaminergic supersensitivity [12], [13], [14], which is unleashed mainly by reduction of DA in the synaptic cleft. Considering the percentage of SWS we observed an increase during the 2^nd^ and 3^rd^ days of recording followed by a return to baseline values after those time-points. Notably, the reduction of the dopaminergic nigral population generated a pronounced potentiation of SWS to the detriment of REM sleep. In addition, sleep efficiency was found to be augmented indicating increased sleepiness during the four first days in the lesioned rats.

We proposed that dopaminergic neurons present in the SNpc possess a fundamental role in the regulation of sleep processes, particularly in promoting REM sleep. It also should be mentioned that flanking areas of the SNpc, like the ventral tegmental area, have recently been reported to be involved in the regulation [16], and perhaps genesis, of REM sleep as we demonstrated for the SNpc. Besides, clinical evidence demonstrate a transient restoration of motor control in PD patients during REM sleep [32], representing, in the light of our study, an intersection between these two physiological functions, with the nigrostriatal pathway playing a key role.

In conclusion, we reported herein that dopaminergic neurons resident in the SNpc possess a central role in the regulation of sleep processes, particularly of REM sleep. This evidence directly demonstrates that the SNpc is an integrative area of biological function, well known to be responsible for voluntary motor control, and herein described as an important center of sleep regulation.
